# Biological and clinical implications of metastasis‐associated circular RNAs in oesophageal squamous cell carcinoma

**DOI:** 10.1002/2211-5463.13297

**Published:** 2021-10-16

**Authors:** Xin Fang, Sachin Mulmi Shrestha, Li‐Hua Ren, Rui‐hua Shi

**Affiliations:** ^1^ Medical College Southeast University Nanjing China; ^2^ Department of Gastroenterology Zhongda Hospital Affiliated Hospital of Southeast University Nanjing China

**Keywords:** circRNA, EMT, metastasis, oesophageal squamous cell carcinoma

## Abstract

Oesophageal squamous cell carcinoma (OSCC) is a prevalent malignancy with high morbidity and mortality as a result of early metastasis and poor prognosis. Metastasis is a multistep process, involving various signalling pathways. Circular RNAs (circRNAs) are a class of covalently closed noncoding RNAs, the aberrant expression of which is reported to be involved in several biological events, including cell transformation, proliferation, migration, invasion, apoptosis and metastasis. Several studies have reported interactions between circRNAs and metastasis‐associated signalling pathways. The abundance, stability and highly specific expression of candidate circRNAs make them potential biomarkers and therapeutic targets in OSCC. In this review article, we comprehensively describe metastasis‐related circRNAs and their interactions with epithelial–mesenchymal transition‐associated molecules. We also describe the molecular mechanisms and clinical relevance of circRNAs in OSCC progression and metastasis.

## Graphical abstract

The aberrant expression of circular RNAs (circRNAs) is reported to be involved in several biological events, including proliferation, cell cycle, apoptosis and metastasis. In this review article, we comprehensively describe metastasis‐related circRNAs and their interactions with epithelial–mesenchymal transition‐associated molecules, as well as the molecular mechanisms and clinical relevance of circRNAs in oesophageal squamous cell carcinoma progression and metastasis.

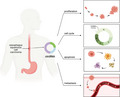

**Abbreviations**
CDC: cell division cycle
circRNA: circular RNA
EMT: epithelial–mesenchymal transition
EC: esophageal carcinoma
FERMT1: fermitin family member 1
Foxo: forkhead box O class
GLUT1: glucose transporter 1
GSK3β: glycogen synthase kinase‐3β
HMGA2: high mobility group AT‐hook 2
ITCH: Itchy E3 Ubiquitin Protein Ligase
LNM: lymph node metastasis
LPAR3: lysophosphatidic acid receptor 3
miRNA: microRNA
MMP: matrix metalloproteinase
NF‐κB: nuclear factor‐κB
NRIP1: nuclear receptor‐interacting protein 1
NTRK2: neurotrophic receptor tyrosine kinase 2
OC: oesophageal cancer
OS: overall survival
OSCC: oesophageal squamous cell carcinoma
PI3K: phosphatidylinositol‐4,5‐bisphosphate 3‐kinase
mTOR: mammalian target of rapamycin
PPAR: peroxisome proliferator‐activated receptor
PTEN: phosphatase and tensin homolog
RBBP7: RB‐binding protein 7
RBP: RNA‐binding protein
TNM: tumour node metastasis
TTC17: tetratricopeptide repeat domain 17
XRCC1: X‐ray repair cross‐complementing 1
ZEB1: zinc‐finger E‐box‐binding homeobox 1

## Oesophageal cancer

Oesophageal cancer (OC) is one of the major challenges to human health, representing the eighth most common malignancy worldwide [1]. Although there has been an increase in age‐standardized 5‐year relative survival and a decline in age‐standardized mortality rates in OC, likely attributed to early screening and detection programmes, as well as increased access to effective treatment over time [2, 3], OC still ranks as sixth among the most common cancer‐related deaths worldwide.

Oesophageal squamous cell carcinoma (OSCC) is the major histological type of OC, constituting approximately 90% of the annual OC incidence [4]. The development of OSCC is a complex process with multiple factors impacting its pathogenesis. For instance, smoking and excess alcohol consumption represent two main environmental risk factors for OSCC [5]. The pathophysiological process of OSCC is typically initiated by recurrent chemical and physical stimulations of the oesophageal epithelial mucosa, which facilitates OSCC progression from accumulating genetic mutations to precursor basal cell dysplasia, and subsequently to invasive carcinoma [6]. Although the molecular mechanisms underlying OSCC tumourigenesis have not been well characterized, the application of high‐throughput sequencing technology has deepened our understanding and indicated common mechanisms regulating OSCC carcinogenesis [7, 8, 9]. Indeed, numerous genetic and epigenetic factors have been investigated, both of which independently, and synergistically, play non‐negligible roles in OSCC carcinogenesis [10]. For instance, somatic mutations of *TP53*, a pivotal gene that affects tumour progression, chromosomal instability and the regulation of metastasis, are corroborated in more than 90% of cases [8, 11]. Moreover, deletions in *CDKN2A*, which controls cell‐cycle arrest, were reported in more than 60% of cases and were found to be associated with stepwise progression from inflammation to cancerous lesions [8, 12]. Other signalling pathways, such as Wnt/β‐catenin pathway regulation, have been frequently demonstrated to possess genetic mutations in various malignancies, including OSCC [13]. Furthermore, epigenetic modifications, such as DNA methylation, are involved in OSCC development [7]. Meanwhile, numerous noncoding RNAs participate in regulating OSCC tumourigenesis [14]. For instance, the long noncoding RNA TTN‐AS1 reportedly promotes OSCC cell proliferation and metastasis through the expression of the transcription factor SNAIL1 by competitively binding to microRNA (miRNA) 133b, resulting in the epithelial–mesenchymal transition (EMT) cascade [15]. Considering that these genomic and epigenomic alterations are associated with the hallmarks of OSCC, we have elected to review the role of circular RNAs (circRNAs) in OSCC.

## circRNA

In the years after the initial discovery of circRNA via electron microscopy in 1976 [16], only sporadic circRNA reports were made because of a lack of reliable approaches. Subsequently, circRNAs were regarded as errant by‐products [17]; however, with the advancements of high‐throughput sequencing, combined with the development of specialized computational pipelines, circRNAs were regarded not as ‘transcriptional noise’ but as a special class of endogenous noncoding RNA in the form of covalently closed rings produced by backsplicing [18]. This special loop structure, with no 5′ end cap structures or 3′ end polyadenylated tails, makes circRNAs insensitive to RNA exonucleases or RNase R nucleic acid exonucleases, and thus more stable than linear RNAs [19].

Recent studies have highlighted the prevalence and highly tissue/cell‐type‐specific expression of circRNAs in OSCC through a combination of bioinformatics and molecular biological methods. For example, differential expression profiles of 861 circRNAs in different tumour and nodal stages were detected via microarray analysis and quantitative RT‐PCR, among which hsa_circRNA_100873 was the most significant candidate with a consistent expression pattern. Patients with early tumour stage, late nodal stage and relatively higher metastatic ability presented different hsa_circRNA_100873 levels compared with the healthy group and the group containing advanced tumour stage with early nodal stage. Moreover, miR‐522‐3p, miR‐1236‐3p, miR‐3064‐5p, miR‐6504‐5p and miR‐943 were predicted to bind to hsa_circRNA_100873. This discrepancy in hsa_circRNA_100873 expression confirmed its function in regulating the invasion and metastasis of OSCC [20]. In addition, elevated levels of circular tetratricopeptide repeat domain 17 (circ‐TTC17) were detected in OSCC cells, plasma and tissues. *In vitro*, cell proliferation and migration increase with circ‐TTC17 expression. Statistical analysis demonstrated that a relatively high circ‐TTC17 expression level is correlated with tumour node metastasis (TNM) stage, lymph node metastasis (LNM) and inferior overall survival (OS) [21]. Moreover, circ‐DLG1 (hsa_circ_0007203) is increasingly expressed in OSCC tissues, as well as in plasma samples with coherently advanced TNM stage. In addition, silencing RNA (siRNA)‐based experiments demonstrated that silencing circ‐DLG1 impedes OSCC cell proliferation [22]. Similar oncogenic circRNAs, such as hsa_circ_0001361, were identified in OSCC tissues, with increased proliferation, migration and invasion abilities and decreased apoptosis in cell lines [23]. Similarly, certain circRNAs exhibited tumour‐suppressive functions in OSCC. For instance, down‐regulation of hsa_circ_0043898 was reported as the most significant among the five screened candidate circRNAs in OSCC specimens and cell lines. The gain‐of‐function assay further verified that hsa_circ_0043898 impairs cell proliferation, migration and invasion while inducing cell apoptosis. Further animal experiments validated the anti‐tumour function of hsa_circ_0043898. RNA sequencing exhibited differential expression of histone H3 and BMI1 in OSCC cell lines [24]. circ‐SMAD7 originates from the backsplicing of *SMAD7* intron 1 on chr18:46470601–46470865. The down‐regulation of circ‐SMAD7 was verified in OSCC plasma samples and specimens and reported as negatively correlated with TNM stage and LNM. Furthermore, the high expression level of circ‐SMAD7 positively affected the proliferation and migration of OSCC cell lines. Although further investigations are required to determine the underlying mechanism, circ‐SMAD7 exhibits potential diagnostic merit in OSCC [25].

Besides tissue/cell‐type‐specific expression, circRNAs also exhibit aberrant expression patterns in exosomes. Exosomes mediate cell‐to‐cell communication and reshape the tumour microenvironment by transporting biomolecules, including circRNAs, thus contributing to tumour growth and metastasis. Hence deciphering the role of exosomal circRNAs would also provide new insights into OSCC. A recent study reported that patients with OSCC with LNM have significantly higher serum exosome has_circ_0026611 levels than patients with OSCC without LNM. Higher expression of has_circ_0026611 is associated with clinical characteristics, such as T and N stages, and postoperative radiotherapy and chemotherapy [26]. Based on the earlier study, hsa_circ_0004771 was also found to be secreted into the extracellular fluid in an exosome‐derived manner. Mechanistic studies showed that hsa_circ_0004771 exhibits proliferative properties both *in vivo* and *in vitro* through promotion of cell‐cycle progression by sponging miR‐339‐5p. Moreover, cell division cycle 25A (CDC25A) was predicted and verified as a downstream targeting molecular signalling protein [27]. Although these two studies demonstrated the presence of circRNAs in exosomes, questions regarding how circRNAs behave through exosomes, and on which target cells, demand further clarification.

The investigation of differentially expressed circRNAs between healthy and cancerous tissues and plasma, and between primary and secondary tumours, sheds light on the promising role of circRNAs as potential OSCC biomarkers and therapeutic targets. In addition, we discuss the clinical relevance of circRNAs and provide a reference point for future studies of OSCC metastatic characterization. Therefore, the aberrant expression of circRNAs has a tumour‐suppressive or oncogenic role that closely correlates with the clinicopathological appearance, prognosis and outcome of OSCC. However, despite the generally accepted correlation between circRNAs and OSCC, the integrated signalling pathways and molecular mechanisms associated with circRNA regulation remain unclear.

### General function patterns of circRNAs in OSCC and other cancers

Several studies have elucidated the versatile functional patterns of circRNAs, from sponging miRNAs to interacting with proteins to regulate host genes, transcription and encoding of proteins. Herein, we review the general biological roles of circRNAs involved in OSCC and summarize those worth investigating in other tumours, with the intent of providing new insights into OSCC research.

#### circRNAs as miRNA sponges in OSCC

It has been documented that circRNAs regulate gene expression via multiple approaches, among which the role of miRNA sponges has been reported most frequently in OSCC. Some circRNAs possess miRNA binding sites and serve as competitive endogenous RNAs to hamper direct base pairing between miRNA and untranslated regions of target mRNA, thus regulating miRNA‐inhibitory gene expression and up‐regulating target gene expression.

Herein, we review the relevant circRNA–miRNA axis (Table 1) and selectively present several mechanisms in which circRNAs regulate OSCC, while discussing the possible shortcomings. Even a single circRNA can bind several miRNAs and exert their function through multiple signalling pathways. The famous circRNA sponge for miR‐7 (CDR1as; also known as ciRS‐7) has been reported to harbour more than 70 conventional miR‐7 binding sites and negatively modulate its expression, thereby regulating the expression of miR‐7 target mRNAs [28, 29]. It was first reported in a study on OSCC that ciRS‐7 could competitively bind miR‐876‐5p by complementary base pairing, resulting in a decrease in the tumour‐suppressive effect of miR‐876‐5p on its downstream target MAGE‐A family. *In vitro*, the ciRS‐7/miR‐876‐5p/MAGE‐A family axis was also corroborated to facilitate tumour growth and metastasis by increasing proliferation markers (Ki67 and PCNA) and metastatic markers (matrix metalloproteinase 2 [MMP2] and MMP9) [30]. Notably, the expression of the downstream MAGE‐A family is associated with high histological grade, LNM and distant metastasis or recurrence. Similarly, the interaction between ciRS‐7 and miR‐7 was confirmed in OSCC by affecting downstream target HOXB13, which mediates p65 phosphorylation, consequently activating the nuclear factor‐κB (NF‐κB) pathway [31]. Recent research has also found that ciRS‐7 up‐regulates matrix metalloproteinase 2 (MMP2) and stem cell marker Kruppel‐like factor‐4, partially by activating the NF‐κB pathway, thereby attenuating miR‐7‐induced invasion in OSCC cells [32]. Overall, ciRS‐7 exerts oncogenic functions through various mechanisms; thus, targeted inhibition might improve current treatment. hsa_circ_0004370, mapped on chr1:170688866–170695542, consists of 358 bases, is up‐regulated in OSCC cells and is associated with tumour size. siRNA‐based experiments verified the pivotal role of hsa_circ_0004370 in modulating oncogenic processing, including promotion of cell proliferation, and invasion, as well as reduced apoptosis via down‐regulating miR‐1294. LIM and SH3 protein 1 has been implicated as tumour activators in the hsa_circ_0004370/miR‐1294 pathway [33]. Further examination of the correlation between hsa_circ_0004370 and clinical entities could provide stronger evidence for the prominent regulatory function of hsa_circ_0004370. Another study reported that hsa_circ_0004370 promotes proliferation, migration and invasion and hinders apoptosis *in vitro* in OSCC by sponging miR‐1301‐3p, further impacting the expression of collagen type I alpha 1 [34]. circRNAs can also exert their function synergistically and antagonistically via different miRNAs through the same signalling pathway. For instance, up‐regulated cZNF292 [35], hsa_circ_0067934 [36], ciRS‐7 [37] and circLAPR3, as well as down‐regulated circVRK1 [38], circLAPR3 [39] and circLAPR4 [40], have been reported in OSCC and further validated to regulate the AKT signalling pathway by sponging their respective miRNAs. Down‐regulated circVRK1 [38], circLAPR4 [40], circular forkhead box O class (circ‐Foxo3) [41] and circPSMC3 [42] all achieved tumour‐suppressive roles by sponging miRNA and subsequently enhancing phosphatase and tensin homolog (PTEN) expression.

**Table 1:** Biological relevance of metastasis‐associated circRNAs in OSCC. Pink: up‐regulating circRNA; green: down‐regulating circRNA. +, promoting effect; −, inhibiting effect; circNELL2, circular neural EGFL‐like 2; circUBAP2, circular ubiquitin‐associated protein 2; COL1A1, collagen type I alpha 1; FNDC3B, fibronectin type III domain containing 3B; HMGB1, high mobility group box 1; KLF‐4, Kruppel‐like factor‐4; LASP1, LIM and SH3 protein 1; NA, not available; NRIP1, nuclear receptor‐interacting protein 1; Paxs, paired box genes; PPAR, peroxisome proliferator‐activated receptor; cZNF292, circRNA zinc‐finger protein 292.

circRNA	Cell proliferation	Migration	Invasion	Apoptosis	Other biological function	Targeting miRNA	Identified targeting gene/pathway	Publication year	Reference
**Up‐regulating circRNA**									
has_circ_0000337 (hsa_circRNA_100872)	+	+	+	NA		miR‐670‐5p		2019	[104]
hsa_circ_0000654	+	+	+	−	Facilitating lung metastasis *in vivo*	miR‐149‐5p	IL‐6/STAT3	2019	[88]
hsa_circ_001275	+	NA	+	−		miR‐370‐3p	Wnt7a	2020	[92]
hsa_circ_0012563	+	+	+	+	Reducing G1 cell‐cycle arrest		XRCC1/EMT	2020	[83]
hsa_circ_0030018	+	NA	+	NA		miR‐599	EMAH/EMT	2019	[84]
hsa_circ_0058063	+	NA	NA	NA	Promoting glucose uptake		GLUT1	2020	[96]
hsa_circ_0003340	+	NA	+	NA		miR‐564	TPX2	2020	[105]
hsa_circ_0004370	+	NA	+	−		miR‐1294	LASP1	2019	[33]
hsa_circ_0004370	+	+	+	−		miR‐1301‐3p	COL1A1	2021	[34]
hsa_circ_0004771	+	NA	NA	NA	Inducing G1 cell‐cycle arrest	miR‐339‐5p	CDC25A	2020	[27]
hsa_circ_0006168	+	+	+	NA	Promoting glycolysis and lactate production	miR‐384	RBBP7/S6K/S6 pathway	2020	[97]
hsa_circ_0006168	+	+	+	NA		miR‐100	mTOR	2019	[106]
hsa_circRNA6448‐14	+	+	+	−		miR‐455‐3p		2020	[107]
hsa_circ_0006948 (ciFNDC3B)	+	+	+	NA		miR‐490‐3p	HMGA2/EMT	2019	[85]
circ_100367	+	+	NA	NA	Promoting EMT and radioresistance	miR‐217	Wnt3/EMT	2019	[87]
hsa_circ_100873								2019	[20]
circRNA_100876	+	+	+	−	Reducing G2/M arrest		EMT	2020	[82]
circRNA ciRS‐7 (CDR1as)	NA	+	+	NA		miR‐7	KLF‐4, NF‐κB/p65	2019	[32]
	+	+	+	NA	Enhancing lung metastasis *in vivo*	miR‐876‐5p	MAGE‐A family	2018	[30]
	NA	NA	NA	NA	Inhibit starvation‐induced or Rapamycin‐induced autophagosome formation *in vivo*	miR‐1299	EGFR/Akt/mTOR	2019	[37]
	NA	NA	NA	NA	Promoting lung metastasis	miR‐7	HOXB13, NF‐κB/p65	2018	[31]
circ‐DLG1	+	NA	NA	NA				2020	[22]
circFNDC3B (hsa_circ_0001361)	+	+	+	−				2018	[23]
circGSK3β (circRNA_103443)	+	+	+	NA			GSK3β/β‐catenin/EMT	2019	[44]
circLPAR3	+	+	+	NA		miR‐375/miR‐433	HMGB1	2020	[108]
circLPAR3	NA	+	+	NA	Promoting lung metastasis *in vivo*	miR‐198	MET/RAS/MAPK and PI3K/AKT	2020	[39]
hsa_circ_0067934 (circ‐PRKCI)	+	+	NA	NA	Reducing G2 cell‐cycle arrest			2016	
	+	NA	NA	NA		miR‐3680‐3p	AKT3	2019	[36]
circPRKCI	+	NA	NA	NA	Inducing G0/G1 cell‐cycle arrest, reducing cell radiosensitivity	miR‐186‐5p	PARP9	2020	[93]
circRAD23B	+	NA	+	NA		miR‐5095	PARP2 and AKT2	2019	[109]
circCNOT6L (circ_0006168)	+	NA	NA	−	Regulating iron metabolism	miR‐384	Fibronectin 1	2020	[98]
circPVT1	+	NA	+	−		miR‐4663	Paxs and PPARs	2019	[110]
circNTRK2	+	+	+	−		miR‐140‐3p	NRIP1, EMT	2020	[86]
circNELL2 (hsa_circ_0025933)	+	+	NA	NA		miR‐127‐5p	CDC6	2020	[111]
circ‐TTC17	+	+	NA	NA				2019	[21]
circUBAP2	+	+	+	NA		miR‐422a	Rab10	2019	[112]
circRNA cZNF292	+	+	+	−		miR‐206	AMPK, PI3K/AKT	2019	[35]
circZDHHC5	+	+	+	NA		miR‐217	ZEB1	2021	[113]
has_circ_0000554	+	+	+	−	Elevated radioresistance	miR‐485‐5p	FERMT1	2021	[94]
circRNA_141539	+		+			miR‐4469	CDK3	2021	[114]
circAKT3	+	+	+			miR‐17‐5p	RHOC and STAT3	2021	[115]
circ_0087378	+	+	+	−		miR‐140‐3p	E2F3	2021	[116]
								2021	
**Down‐regulating circRNA**									
hsa_circ_001946	−	−	−	NA		miR‐7‐5p		2019	[117]
hsa_circ_0043898	−	−	−	+	Accelerating cell cycle			2018	[24]
circ‐Foxo3	−	−	−	+	Inducing G0/G1 cell‐cycle arrest	miR‐23a	PTEN	2019	[41]
circPSMC3	NA	NA	NA	+	Increasing cell sensitivity to gefitinib	miR‐10a‐5p	PTEN	2020	[42]
cir‐ITCH	−	NA	NA	NA	Accumulating cells in G1 phase	miR‐17, miR‐7, miR‐214	inhibiting Wnt/β‐catenin	2015	[48]
circLARP4	−	−	NA	+		miR‐1323	PTEN/PI3K/AKT	2020	[40]
circ‐SMAD7	−	−	NA	NA				2019	[25]
circVRK1	−	−	NA	NA	Increasing radiosensitivity of OSCC cells, reversing EMT progress	miR‐624‐3p	PTEN/PI3K/AKT	2019	[38]

All yellow shades indicate that corresponding circRNAs are EMT‐related.

Luciferase reporter gene experiments and functional rescue validation have contributed to the identification of a growing number of circRNAs shown to function through a sponge mechanism. However, most circRNA mechanistic studies are relatively independent, neglecting the fact that various circRNAs and miRNAs exist in OSCC. Due to the complex interconnected networks of circRNAs and miRNAs, integration analysis between circRNAs may lead to new research directions for OSCC to better characterize the relationships between circRNAs. Nonetheless, the interconnected mechanism of circRNA–miRNA sponging confirms that this network plays a role in oesophageal oncogenic expression by interacting with a single common pathway or multiple pathways. Hence the potential regulatory role of the circRNA–miRNA target gene axis may serve as a promising tool for cancer diagnosis and therapy.

#### circRNA interaction with proteins in OSCC

Apart from sponging miRNAs, circRNAs can also interact with other cellular components, such as proteins, to activate or inhibit downstream signalling pathways that affect the malignant behaviour of tumours [43]. Circular glycogen synthase kinase‐3β (circGSK3β) is transcribed from exons 3, 4 and 5 of the GSK3β gene. The parental protein GSK3β is an important negative mediator of the Wnt signalling pathway that regulates the progression of many malignancies, including OSCC [44]. circGSK3β directly binds the N terminus of the parental protein GSK3β and inhibits its activity, leading to the attenuation of GSK3β‐induced β‐catenin phosphorylation, along with sequential ubiquitination and degradation. Therefore, circGSK3β promotes the progression of OSCC by enhancing migration and invasion activity, as well as EMT, via direct interaction with GSK3β. The circGSK3β/GSK3β/β‐catenin network provides a novel mechanism by which circRNAs modulate the activity of parental proteins that directly interact with the parental gene in OSCC progression and metastasis.

#### circRNAs as a regulator of host gene in OSCC

Because most circRNAs are derived from protein‐coding genes and spliced cotranscriptionally [45, 46], it is plausible to assume that the processing of circRNAs can affect the splicing of their cognate transcripts [47]. cir‐ITCH derives from the Itchy E3 ubiquitin protein ligase (ITCH) gene, which has been reported to degrade the phosphorylated form of dishevelled (Dvl) through the proteasome, thereby inhibiting the Wnt signalling pathway. miR‐216b, miR‐17, miR‐214, miR‐7 and miR‐128 were verified to possess a binding affinity to cir‐ITCH. Hyperexpression of cir‐ITCH was substantiated in OSCC specimens and substantially increased cognate linear ITCH expression in constructed cir‐ITCH cells. The correlation between cir‐ITCH and linear ITCH was examined, and it was hypothesized that cir‐ITCH increases the expression of linear ITCH by sponging miRNA. However, no validation of the direct binding sites was provided. Although this study revealed that cir‐ITCH decreased cell proliferation, arrested cells in the G1 phase *in vitro* and repressed cell growth *in vivo* via the cir‐ITCH/Wnt network, the relationship between cir‐ITCH and ITCH requires further validation [48]. circRNAs can also directly bind to their parental gene locus, subsequently leading to transcriptional alterations [49]. However, this mechanism has not been studied in‐depth in OSCC, and more relevant RNAs are worth exploring.

### Other general function patterns of circRNAs

Apart from sponging miRNAs, circRNAs can also target other cellular components, such as RNA‐binding proteins (RBPs) [43]. Through binding, storing or sequestering molecules, circRNAs serve as dynamic scaffolds or decoys in particular subcellular locations. The interaction of circRNAs with RBPs competitively suppresses the binding between the corresponding mRNA and RBPs, subsequently altering gene expression outcomes [50].

Despite the earlier‐mentioned noncoding functions, increasing evidence indicates that circRNAs may not be a true class of noncoding RNAs, with at least a small subset of them being translatable [51], via sequences that act as internal ribosome entry sites [52] or by m6A modification [53]. However, no coding circRNAs have been reported in the OSCC field, probably because of the low abundance of circRNAs compared with linear transcripts and the fact that there are very few circRNAs that possess internal ribosome entry sites and m6A‐modifying sites.

Based on the aforementioned functional patterns and accumulating evidence of the distinct expression profile of circRNAs in OSCC, it can be deduced that circRNAs are implicated in OSCC development and metastasis‐related cellular events. Hence investigation of metastasis‐associated circRNAs may improve molecular and cellular insights into OSCC progression and metastasis. To date, hundreds of circRNAs have been implicated in metastasis‐related processes, including EMT. EMT, the transition from epithelial to mesenchymal states, has been identified as the main promoter and requisite for metastasis. EMT‐related markers can be detected at the invasive front of the tumour and further elaborated by mechanistic studies to drive the dissemination of tumour cells and initiate metastasis [54, 55], which indicates that EMT is sufficient for this process.

Herein, we review the current research on circRNAs related to the metastasis of OSCC while emphasizing the regulatory functions and mechanisms of circRNAs that contribute to the development of malignant phenotypes of OSCC.

## Metastasis

Metastasis, as the end product of a multistep cell biological process involving the dissemination of tumour cells from the primary site to distant sites, not only is the main cause of cancer‐related deaths but also represents a significant challenge to humans successfully fighting cancer [56]. One hundred years ago, the English surgeon Stephen Paget drew an analogy between the metastatic process and botanic seeding [57]. This hypothesis has been widely accepted and evolved. Investigations corroborated that the seed (progenitor cell) grows in congenial soil (the metastatic microenvironment) only when the seed is subsequently adapted to the soil [58]. However, complex metastasis might be simplified into the following steps: (a) local invasion and intravasation, that is, invasion into adjacent tissue and vessels; (b) dissemination to target organs, including survival in the circulation, arrest and extravasation; (c) and colonization, that is, accommodating to a new microenvironment, escaping from apoptosis and resuming proliferative ability, among which proliferation, angiogenesis, invasion, migration and apoptosis are indispensable molecular underpinnings for metastasis [59]. Various genes and corresponding signal pathways underlie the biological interaction between ‘seed and soil’. For instance, EMT, as an imperative process across metastasis, enables cells to detach from their neighbours and gain mesenchymal traits and stem cell‐like properties that favour invasion. Research suggests that less than 0.02% of tumour cells can survive in the bloodstream because of the threat of high shearing forces in the circulation, anoikis‐mediated cell death, immune attack and metastasis [60, 61, 62]. However, specific stem cell‐like characteristics, including up‐regulation of stem cell‐associated genes, self‐renewal properties, high proliferative capacity and the capability of prolonged quiescence, help tumour cells to successfully metastasize. In addition, self‐renewal can be acquired after undergoing EMT via activating signalling pathways, such as AKT [63, 64], which further favours tumour cell survival. AKT reportedly plays an essential role in the proliferation and survival of disseminated tumour cells, regardless of whether they are within the circulation or bone marrow [65]. Transdifferentiation in EMT is also mediated by the phosphatidylinositol‐4,5‐bisphosphate 3‐kinase (PI3K)/AKT/mammalian target of rapamycin (mTOR) pathways [66]. Hypoxia can up‐regulate hypoxia‐inducible factor 1 alpha subunit and zinc‐finger E‐box‐binding homeobox 1 (ZEB1) and further induce EMT [67, 68, 69]. Studies have demonstrated that multiple signal transduction pathways, such as Wnt/β‐catenin and Notch, have a remarkable influence on EMT [70, 71]. Although protein‐coding genomes have attracted a significant proportion of the research community attention, noncoding genes and RNAs comprise the major fractions of genomes and transcriptomes, because less than 2% of the human genome expresses protein‐coding RNAs [72]. More recently, investigations concerning regulation of the metastasis process by noncoding RNAs have provided new insights into the mechanisms of EMT. miRNAs regulate EMT by selectively binding to mRNAs, consequently affecting their translation or degradation [73]. The up‐regulation of miR‐106b in OSCC promotes EMT by targeting PTEN [74]. Emerging evidence has shown that many circRNAs are also involved in the regulatory network of EMT by interacting with various components and adapters, as well as dysregulation of key signalling pathways, such as Wnt, PTEN and AKT [38, 75, 76]. Notably, these interactions further function in the aberrant acquisition of stem cell signalling. The convergence of signalling transduction pathways together modulates EMT, which endows progenitor cells with stemness and epithelial plasticity, and enhances tumour progression and resistance to chemotherapy [77, 78].

**Fig. 1:**
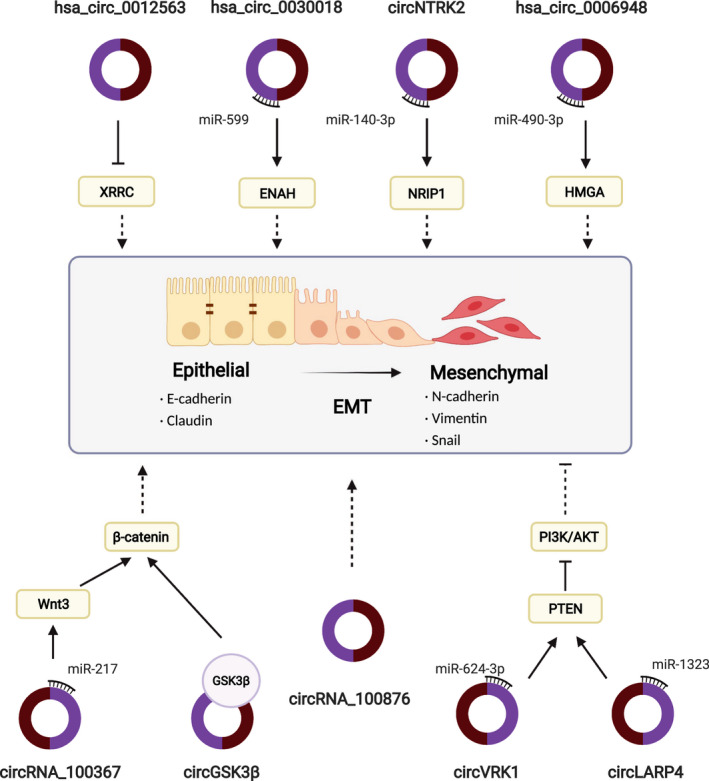
Interconnected regulatory networks of circRNAs and EMT in OSCC. Several circRNAs in OSCC (has_circ_0030018, circNTRK2, has_circ_0006948, has_circ_100367, circVRK1 and circLARP4) play a role on the EMT pathway via the circRNA/miRNA/mRNA axis by expressing different epithelial and mesenchymal protein markers. hsa_circ_0012563 increases the expression of XRCC1, consequently enhancing the abundance of the EMT marker E‐cadherin and increasing N‐cadherin expression. circGSK3β promotes the EMT process via direct interaction with parental protein GSK3β and inhibition of β‐catenin phosphorylation. hsa_circRNA_100876 decreases the protein level of epithelial–phenotype E‐cadherin and reduces mesenchymal–phenotype N‐cadherin, vimentin and the EMT‐associated transcription factor SNAIL. This figure was created with BioRender.com.

In view of the regulatory role of circRNA in metastatic pathways, it may be clinically applicable as a diagnostic and therapeutic tool. Indeed, features of circRNAs, such as abundance, conservation, stability, and tissue‐ and developmental stage‐specific expression, indicate the promising role of circRNA as a biomarker in cancer. Studies have shown that circRNA can be applied to evaluate the effect of diagnosis and treatment and to predict the prognosis and metastasis of many diseases, including OSCC [20, 79, 80, 81]. Several circRNAs have been shown to be correlated with metastatic clinicopathological characteristics (Table 2). Because metastasis is a major detrimental cause of cancer‐related death, it is important to explore the mechanisms of circRNA function and its relevance to metastasis. In this article, we review the current research and emphasize the biological role of circRNAs as metastasis regulators by interacting with EMT‐associated factors in OSCC (Fig. 1). We also review metastasis‐associated circRNAs in OSCC while focusing on the mechanisms by which circRNAs function and regulate OSCC metastatic features of OSCC. In addition, we review metastasis‐associated circRNAs and discuss potential EMT regulatory networks in OSCC. Therefore, we investigate the practical challenges associated with these studies and how they might be overcome.

**Table 2:** Summary of circRNAs that are identified as biomarkers in OSCC. DFS, disease‐free survival; FNDC3B, fibronectin type III domain containing 3B; MFS, metastasis‐free survival; RFS, recurrence‐free survival.

circRNA name	Sample	Clinical application	Clinical relevance	Reference
**Up‐regulating circRNA**				
hsa_circ_0000654	55 pairs of OSCC and adjacent tissues	Prognostic indicator	Correlated with the higher T stage and local LNM	[88]
hsa_circ_0012563	60 pairs of OSCC and adjacent normal tissues	Prognostic indicator	Associated with metastasis and lower OS	[83]
hsa_circ_0004370	25 pairs of OSCC and adjacent normal tissues	Risk predictor	Associated with the tumour size	[33]
	50 pairs of OSCC tissues and nearby healthy oesophageal tissues		TNM stage tumour size	[34]
Exosome hsa_circ_0004771	20 pairs tissues and 105 pairs of case–control plasma, 6 paired preoperative and postoperative plasma	Risk predictor and prognostic indicator	Correlated with T grade, vascular invasion, and shorter OS, DFS	[27]
hsa_circ_0006168	52 pairs of OSCC and normal tissues	Risk predictor	Associated with the TNM stage and LNM	[106]
hsa_circRNA6448‐14	82 pairs of OSCC and normal tissues	Diagnostic marker and prognostic marker	Correlated with worse differentiation, advanced pTNM stage, worse DFS and OS	[107]
hsa_circ_0006948 (ciFNDC3B)	153 pairs of OSCC and adjacent normal tissues	Independent prognostic factor	Associated with LNM and shorter OS	[85]
circRNA_100876	74 pairs of OSCC and adjacent normal tissues	Risk predictor and prognostic indicator	Correlated with tumour invasion depth, LNM, and vascular invasion, negatively correlated with survival outcome (RFS OS)	[82]
ciRS‐7	86 pairs of OSCC and adjacent normal tissues, 112 cases of primary OSCC tissues, 244 cases of lymph node tissues, and 26 cases of distant metastatic tissues	Risk predictor	Correlated with pathological grade and clinical stage	[30]
	123 pairs of OSCC and normal tissues	Risk predictor and independent prognostic indicator	Correlated with advanced TNM stage, shorter OS, DFS	[31]
circ‐DLG1 (hsa_circ_0007203)	55 pairs of OSCC and normal tissues, and 63 plasma (28 healthy, 35 OSCC)	Risk predictor	Associated with TNM stage	[22]
circGSK3β (circRNA_103443)	50 pairs of OSCC and normal tissues, and 10 pairs of preoperative and postoperative plasma	Risk predictor and prognostic indicator	Positively associated with advanced clinical stage, shorter MFS and OS	[44]
circLPAR3	50 pairs of OSCC and normal tissues and peripheral blood samples, and 31 healthy peripheral blood samples	Risk predictor and prognostic indicator	Positively correlated with N classification and TNM stage and shorter OS	[108]
circLPAR3	52 pairs of OSCC and paracarcinoma tissues	Risk predictor	Correlated with LNM and advanced TNM stage	[39]
hsa_circ_0067934	51 pairs of OSCC and normal tissues	Risk predictor and prognostic marker	Correlated with poor differentiation, T stage and TNM stage	[36]
circNTRK2	56 pairs of OSCC and adjacent normal tissues	Risk predictor and prognostic marker	Correlated with advanced TNM stage, LNM and shorter OS	[86]
circ‐SLC7A5	23 pairs of OSCC and corresponding tissues, 87 OSCC plasma and 53 normal bloods	Circulating biomarker and prognostic indicator	Associated with TNM stage and inferior survival time	[118]
circ‐TTC17	25 pairs of OSCC and corresponding nontumorous tissues, 25 normal plasma and 30 OSCC plasma	Diagnostic marker and prognostic marker	Positively associated with TNM stage, lymphatic metastasis and inferior OS	[21]
hsa_circ_0026611	69 serum samples from patients with OSCC, 35 patients with LNM and 34 patients without LNM	Diagnostic marker and prognostic marker	Correlated with T stage, N stage, postoperative radiotherapy and chemotherapy, LNM and shorter OS	
circRNA_141539	50 pairs of OSCC tissues and adjacent noncancer tissues		Associated with TNM stage, T stage and N stage and negatively associated with histological grade	[114]
circAKT3	82 OSCC tissue sample		Tumour size, lymphatic metastasis and clinical TNM staging were positively related	[115]
circ_0087378	50 pairs OSCC and normal tissues		Positively associated with tumour size, histological grade, tumour stage, the presence of metastasis and worse survival	[116]
**Down‐regulating circRNA**				
hsa_circ_001946	50 pairs of tumour and nontumour tissues and 100 plasma (50 healthy and 50 OSCC patients)	Diagnostic marker and prognostic marker	Associated with DFS and OS	[117]
circ‐Foxo3	94 pairs of OSCC and adjacent tissues	Risk predictor	Associated with TNM stage	[41]
circ‐SMAD7	36 pairs of OSCC and adjacent normal tissues, 32 OSCC plasma and 25 healthy plasmas	Risk predictor	Negative correlation with TNM stage and LNM	[25]
circVRK1	88 pairs of OSCC and adjacent nontumour tissues	Prognostic indicator	Correlated with higher OS	[38]

Pink shades stand for up‐regulated circRNAs while green shades means down‐regulated circRNAs.

hsa_circRNA_100876 has been reported as up‐regulated in OSCC samples and contributes to the acceleration of tumour progression both *in vivo* and *in vitro*. In addition, circRNA_100876 enhances migration, invasion and EMT processes, while eliminating cell‐cycle arrest and inhibiting apoptosis. Silencing circRNA_100876 increases the protein level of epithelial–phenotype E‐cadherin and reduces mesenchymal–phenotype N‐cadherin, vimentin and the EMT‐associated transcription factor SNAIL. Clinical data have further shown that high expression of circRNA_100876 strongly correlates with multiple metastatic indicators, such as tumour invasion depth (*P* = 0.017), LNM (*P* = 0.027) and vascular invasion (*P* = 0.036) [82].

Other *in vitro* studies have revealed a mechanism underlying the regulatory role of circRNA in metastasis. Higher hsa_circ_0012563 expression was validated in OSCC in both clinical specimens and OSCC cell lines and was shown to be positively correlated with a lower survival rate and metastasis. *In vitro*, hsa_circ_0012563 positively affects cell proliferation, migration, invasion and apoptosis. Moreover, siRNA‐based experiments demonstrated that X‐ray repair cross‐complementing 1 (XRCC1) is the downstream molecule, and silencing of hsa_circ_0012563 increases the expression of XRCC1, consequently increasing the abundance of the EMT marker E‐cadherin and inhibiting N‐cadherin expression. Meanwhile, repression of XRCC1 also partly neutralizes the inhibitory effect on E‐cadherin protein expression and promotes the effect on N‐cadherin protein expression [83]. In addition, up‐regulation of hsa_circ_0030018 was observed in OC cells. Molecular experiments revealed that hsa_circ_0030018 can positively regulate cell proliferation, migration and the EMT process by functioning as an endogenous miR‐599 sponge to inhibit miR‐599 activity, which consequently up‐regulates enabled homolog expression. Meanwhile, hsa_circ_0030018 depletion increases E‐cadherin protein levels and decreases that of N‐cadherin and vimentin. Down‐regulation of enabled homolog restores the protein expression of E‐cadherin, N‐cadherin and vimentin [84]. Similarly, hsa_circ_0006948 is up‐regulated in OSCC and is associated with poor survival and lymphatic metastasis. *In vitro*, hsa_circ_0006948 promotes proliferation, migration and invasion capacity and induces EMT. In addition, miR‐490‐3p was found to bind to hsa_circ_0006948. The high mobility group AT‐hook 2 (HMGA2) was reported to facilitate EMT before it was verified to be a direct target of hsa_circ_0006948/miR‐490‐3p and affect the expression of vimentin, N‐cadherin and E‐cadherin. Thus, hsa_circ_0006948 could directly bind miR‐490‐3p to regulate HMGA2, further inducing EMT [85]. hsa_circ_0026231 is derived from the gene *La ribonucleoprotein 4* (*LARP4*) with a length of 1313 nucleotides. The functional relevance of circLARP4 is primarily exhibited in hindering cell proliferation and migration, driving apoptosis and impeding the EMT process by affecting the protein levels of E‐cadherin and N‐cadherin. circLARP4 inhibits miR‐1323 activity, thereby modulating the PTEN‐mediated PI3K/AKT pathway to impact the EMT process and other biological behaviours of OSCC cells [40]. circRNA neurotrophic receptor tyrosine kinase 2 (circNTRK2) is located on chr9:87356806–87367000 with a length of 237 bp. Clinical data also demonstrated that up‐regulation of circNTRK2 is associated with TNM stage, LNM and worse OS. Functionally, circNTRK2 facilitates cell proliferation, invasion and EMT in OSCC *in vitro* and promotes tumour growth *in vivo*. By sponging miR‐140‐3p, circNTRK2 further up‐regulated the expression of nuclear receptor‐interacting protein 1, which is related to various oncogenic pathways, thereby exerting its oncogenic role [86].

Interestingly, research has shown that OSCC radioresistant cell lines possess stronger molecular characteristics with lower E‐cadherin expression and higher vimentin and SNAIL expression. The level of hsa_circRNA_100367 is also elevated in this radioresistant cell line and is positively associated with EMT. Meanwhile, silencing hsa_circRNA_100367 enhances the expression of EMT‐related molecules, such as vimentin and SNAIL, through the hsa_circRNA_100367/miR‐217/Wnt3 axis [87]. Similarly, elevated levels of circVRK1 attenuate cell proliferation, migration and EMT and reinforce cell sensitivity to radiotherapy in OSCC cells. By sponging miR‐624‐3p, circVRK1 up‐regulates PTEN and subsequently inactivates the downstream PI3K/AKT signalling pathway, thereby altering EMT [38].

The studies discussed earlier focus on the relationship between circRNAs and metastasis by detecting the protein levels of EMT markers *in vitro*. Besides the alteration of EMT markers, circRNA can also directly facilitate the metastasis process *in vivo*. High expression of circ_0000654 was first detected in OSCC specimens and confirmed to be significantly associated with an increased T stage and local LNM in patients with OSCC. *In vitro*, circ_0000654 was found to accelerate OSCC progression by adsorbing miR‐149‐5p and indirectly activating the IL‐6/STAT3 signalling pathway. In addition, a lung metastasis model was constructed through vein injection, and hematoxylin and eosin staining revealed that knockdown of circ_0000654 significantly reduces lung metastasis with less OSCC cell infiltration [88]. However, the results might be more convincing if shRNA instead of siRNA was applied in the xenograft model because shRNA exhibits a longer silencing effect. Another study claimed that circular lysophosphatidic acid receptor 3 (circLPAR3), which arises from the single cyclization of exon 2 on the *LPAR3* gene, could promote migration, invasion and metastasis *in vivo* and *in vitro*. However, simple alterations in protein levels of transfer‐associated molecules, such as MMP2 and MMP9, in the sh‐circLPAR3 group alone are not sufficient to account for the prometastatic effect of circLPAR3 *in vitro*, and more direct evidence is required to verify this inference.

## Potential therapeutic targets for OSCC

### Facilitation of tumour resistance to chemotherapy and radiotherapy

Treatments for OSCC have been developed to cure or delay the disease, including endoscopic therapy, surgical resection, chemotherapy, chemoradiotherapy and neoadjuvant chemoradiotherapy. Notably, the alliance of neoadjuvant chemoradiotherapy and surgery benefits patients with OSCC with curable potential [89]; nevertheless, approximately 60% of patients show no response to CCRT [90]. Moreover, due to intratumour heterogeneity and tumour variability, the heterogeneity of resistance affects the chemotherapeutic effect [91]. circRNAs were observed to exhibit a potential role in the chemotherapy treatment of OSCC. Higher expression of circRNA_001275 was observed in pathologically confirmed cisplatin‐resistant tissue and cell lines compared with adjacent cisplatin‐sensitive tissue and regular OC cell lines. The protumour function of circRNA_001275 was exerted through the circRNA_001275/miR‐370‐3p/Wnt7a regulatory network. circRNA_001275 was shown to enhance cell viability and invasiveness and inhibit apoptosis. However, further investigation with cisplatin interference is required to validate the proresistance effect of circRNA_001275 [92].

Because circRNAs are expressed differently in drug‐resistant tissues and cell lines, it would be reasonable to speculate on their role in OSCC tumour resistance. In fact, a study reported the down‐regulation of circPSMC3 in OSCC tissues and gefitinib‐resistant cell lines. Vector overexpression of circPSMC3 significantly increased cellular sensitivity to gefitinib, with a lower survival rate of OSCC cells and corresponding gefitinib‐resistant cells, and lower half‐maximal inhibitory concentration value of gefitinib than the control group. circPSMC3 also accelerated apoptosis, with high cleaved caspase‐3 protein expression. The underlying mechanism of circPSMC3 was also explored, showing that circPSMC3 down‐regulates PTEN by acting as an miR‐10a‐5p sponge to suppress the progression of OSCC, meaning that the circPSMC3/miR‐10a‐5p/PTEN axis may be a reliable treatment strategy in the future [42]. ciRS‐7 was also found to have a regulatory role in starvation or rapamycin‐induced autophagy in OSCC cell lines. Knockdown of ciRS‐7 significantly increases the number of autophagosomes, while its overexpression significantly decreases the levels of autophagy markers LC3 and P62. In addition, silencing ciRS‐7 strengthens LC3 and p62 degradation. The miR‐1299/EGFR/Akt/mTOR axis was verified as a downstream mechanism in starvation or rapamycin‐induced autophagy in OSCC [37].

circRNAs also have the potential to modulate the radioresistance of OSCC. circPRKCI is formed by cyclization of exons 15 and 16 of the *PRKCI* gene, with 1484 bp in length. Flow cytometry and western blotting revealed that inhibited circPRKCI expression augments cell apoptosis with an increase in radiation dose, while colony formation assay results revealed that PARP9 enhancement overturns the circPRKC inhibition‐mediating effect on cell radiosensitivity. circPRKCI affects the anti‐radioresistant phenotypes *in vitro* by sequestering miR‐186‐5p and further activating PARP9 [93]. Collectively, circPRKCI may serve as a potential target for treating OSCC.

Elevated levels of circVRK1 attenuate cell proliferation, migration and EMT and reinforce cell sensitivity to radiotherapy. Overexpression of miR‐624‐3p and inhibition of PTEN reverse the radiosensitivity enhancement induced by circVRK1. Hence circVRK1 suppresses OSCC radioresistance via the miR‐624‐3p/PETN axis [38]. A similar study reported that hsa_circ_0000554 is up‐regulated in OSCC radioresistant specimens. Cell colony formation showed that knockdown of circ_0000554 represses the cell survival fraction while increasing caspase‐3 activity under radiation. The colony formation assay showed that fermitin family member 1 (FERMT1) overexpression attenuates the down‐regulation of the circ_0000554‐mediating effect on cell radiosensitivity. Further experiments validated that circ_0000554 promotes progression and elevated radioresistance through the miR‐485‐5p/FERMT1 axis [94]. hsa_circRNA_100367 is also highly expressed in radioresistant OC cell lines, whereas silencing hsa_circRNA_100367 enhances radiation sensitivity. circRNA_100367 also positively affects cell proliferation and migration under the radiation dose. Nude mice were divided into four groups and inoculated with OSCC, radioresistant OSCC, stably high‐circRNA_100367 level OSCC and low‐circRNA_100367 level radioresistant OSCC cells, which were irradiated 10 days after injection. The high‐circRNA_100367 group showed significantly larger tumour volume than the control group, while the low‐circRNA_100367 group had tumours with smaller volumes, indicating that circRNA_100367 can promote radioresistance both *in vitro* and *in vivo*.

### Regulating tumour metabolism

One of the common features of tumour cells is that they proliferate indefinitely. To sustain rapid growth and progression, altered metabolism is essential to provide more nutrition to the tumours [95]. Emerging studies have highlighted the regulatory role of circRNAs in tumour metabolism. The circ_0058063 level is increased in OSCC and positively correlates with glucose transporter 1 (GLUT1) mRNA level and protein expression. Downstream function analysis verified that circ_0058063 enhances OSCC proliferation by up‐regulating GLUT1 to increase glucose uptake [96]. Nonetheless, the mechanism underlying the positive regulation of GLUT1 by circ_0058063 remains obscure. Future studies are required for this novel therapeutic target.

Similarly, knockdown of hsa_circ_0006168 inhibits glucose consumption and lactate production *in vitro* by down‐regulating pyruvate kinase. Loss‐of‐function experiments were conducted to further explore the relationship between hsa_circ_0006168, miR‐384 and RB‐binding protein 7 (RBBP7), and hsa_circ_0006168 was found to facilitate cell growth, migration, invasion and glycolysis by regulating miR‐384/RBBP7. In addition, the S6K/S6 pathway becomes activated by up‐regulating RBBP7 induced by hsa_circ_0006168 to modulate OC progression [97]. Lending credence to the earlier two studies, circ_0006168 (also termed circCNOT6L in this study) was also validated to bind to miR‐384 to increase FN1 (fibronectin 1) expression. In addition, circCNOT6L affects the iron metabolism of OSCC cells by altering the protein levels of iron regulator proteins 1 and 2, transferrin receptor and ferritin light chain [98].

## Discussion

Manifold literature has deepened our understanding of circRNAs and their relevance to various physiological and pathological processes. The intricate mechanisms underlying multitargeting circRNAs are associated with the initiation, metabolism, metastasis and other pathological OSCC processes. However, some obstacles still need to be overcome for future studies and application.

From the detection aspect, the earlier‐mentioned studies have been based on second generation sequencing and focused on exon‐derived circRNAs. However, many issues are associated with short‐read RNA sequencing, in particular the inability to confirm the full‐length sequence and accurately quantify the circRNA. Meanwhile, novel detection methods, such as nanopore technology, catalogue full‐length circRNA with higher accuracy for circRNAs with low expression and mitochondrial circRNAs [99]. Long‐read sequencing may facilitate identification of additional alternative splicing events within the circRNAs, including more retained intron events, that will ultimately provide new insights on OSCC research [100].

From the validation aspect, it is important to clarify circRNA localization because its function is highly dependent on its space specificity. Select studies have leveraged fluorescence *in situ* hybridization to provide intracellular localization data in OSCC, whereas none of the earlier‐discussed studies provided *in situ* data to assess potential intratumour heterogeneity related to the expression of circRNAs. A recent study found that ciRS‐7 is abundantly expressed in tumour stromal cells within the tumour microenvironment and is completely absent in colon cancer cells [101]. The implication that different tumour/stroma ratios affect the validation of circRNA revealed new insights in OSCC. The strategies of cell sorting and chromogenic RNA *in situ* hybridization could be applied to clarify the cellular expression patterns of circRNA more adequately in OSCC and exclude the influence of matrix on the results.

In addition, predominant mechanistic studies of circRNAs in OSCC focus on their role as miRNA sponges in modulating downstream molecules, because most circRNAs are localized in the cytoplasm. However, one circRNA possesses multiple miRNA binding sites. Thus, focusing on one miRNA might neglect that some miRNAs have opposite downstream effects. It is thus important to determine whether circRNAs act through several miRNAs simultaneously, or through one primary miRNA when in conditions with very low levels of additional miRNA binding sites. Moreover, other general function patterns, such as the interaction between proteins and DNA, have rarely been investigated in OSCC and deserve more attention [102].

Because circRNAs can alter the characteristics of tumours, several factors can lead to altered levels of circRNA. It was reported that ZEB1, as a key transcription factor, could bind the promoter region of hsa_circ_0001178 and increase its expression in colorectal cancer [103]; similar studies that explore the upstream trigger of alternative splicing, which generate aberrant circRNAs, are needed in OSCC.

Furthermore, the development of OSCC is a complex process with multiple stages and successive activation of various cytokines and mechanisms. Investigations focusing on the aberrant expression of primary and metastatic OSCC tissue might provide more holistic evidence of the role of circRNAs in different periods of metastasis. In addition, although circRNAs are expected to assist in clinical diagnosis, prognosis and treatment according to current data, a single study may not be comprehensive and sufficiently in‐depth to verify whether circRNA could serve as a therapeutic target and as an upstream regulator. Further evidence, especially via clinical trials, is required.

## Conclusions

In summary, OSCC is a multistage and multifactorial disease with a mechanism that has not been fully elucidated. The lack of early typical clinical symptoms leads to late diagnosis and poor prognosis in patients with advanced OSCC, particularly in patients with distant metastasis. circRNAs as novel noncoding RNAs have been shown to mediate various biological events, including cell transformation, proliferation, migration, invasion, apoptosis and metastasis in OSCC. The aberrant expression of circRNAs was substantiated in OSCC and correlated with multiple clinicopathological features, including metastasis. In addition, various circRNAs play crucial roles in the development, metastasis and resistance to chemotherapy and radiotherapy in OSCC. In this regard, circRNAs exhibit promising value in clinical diagnostic, prognostic and therapeutic applications. Identifying an increasing number of circRNAs that affect OSCC is important, while exploring deeper triggers of alternative splicing that generate aberrant circRNAs might provide new insights into OSCC. Therefore, investigations on the regulation of OSCC by circRNAs are still in their infancy, and future mechanistic studies together with clinical research appear to be a future endeavour.

## Conflict of interest

The authors declare no conflict of interest.

## Author contributions

XF and SMS conceived and designed the project. XF acquired, analysed and interpreted the data. XF wrote the manuscript. XF, SMS and L‐HR made manuscript revisions. RS organized the research project, reviewed the manuscript and arranged necessary resources from the grants. All authors approved the final version of the manuscript.
